# Comparative Analysis of 37 *Acinetobacter* Bacteriophages

**DOI:** 10.3390/v10010005

**Published:** 2017-12-24

**Authors:** Dann Turner, Hans-Wolfgang Ackermann, Andrew M. Kropinski, Rob Lavigne, J. Mark Sutton, Darren M. Reynolds

**Affiliations:** 1 Department of Applied Sciences, Faculty of Health and Applied Sciences, University of the West of England, Coldharbour Lane, Bristol BS16 1QY, UK; Darren.Reynold@uwe.ac.uk; 2 Faculty of Medicine, Department of Microbiology, Immunology and Infectiology, Université Laval, Quebec, QC G1X 46, Canada; 3 Departments of Food Science, Molecular and Cellular Biology; and Pathobiology, University of Guelph, Guelph, ON N1G 2W1, Canada; Phage.Canada@gmail.com; 4 Laboratory of Gene Technology, KU Leuven, Kasteelpark Arenberg 21, box 2462, 3001 Leuven, Belgium; Rob.Lavigne@kuleuven.be; 5 National Infections Service, Public Health England, Porton Down, Salisbury, Wiltshire SP4 0JG, UK; Mark.Sutton@phe.gov.uk

**Keywords:** *Acinetobacter baumannii*, *Acinetobacter* phages, bacteriophages, bioinformatics, Comparative genomics, protein clustering, phylogeny

## Abstract

Members of the genus *Acinetobacter* are ubiquitous in the environment and the multiple-drug resistant species *A. baumannii* is of significant clinical concern. This clinical relevance is currently driving research on bacterial viruses infecting *A. baumannii*, in an effort to implement phage therapy and phage-derived antimicrobials. Initially, a total of 42 *Acinetobacter* phage genome sequences were available in the international nucleotide sequence databases, corresponding to a total of 2.87 Mbp of sequence information and representing all three families of the order *Caudovirales* and a single member of the *Leviviridae*. A comparative bioinformatics analysis of 37 *Acinetobacter* phages revealed that they form six discrete clusters and two singletons based on genomic organisation and nucleotide sequence identity. The assignment of these phages to clusters was further supported by proteomic relationships established using OrthoMCL. The 4067 proteins encoded by the 37 phage genomes formed 737 groups and 974 orphans. Notably, over half of the proteins encoded by the *Acinetobacter* phages are of unknown function. The comparative analysis and clustering presented enables an updated taxonomic framing of these clades.

## 1. Introduction

Bacterial viruses (bacteriophages) are considered to be the most prevalent entities in the biosphere [1]. Both lytic and temperate phages act as architects of bacterial evolution and diversification through predation and the facilitation of horizontal gene transfer [2]. The genomic diversity of the bacteriophages appears to be immense and has been proposed to represent the largest source of gene diversity in the natural world, a feature emphasised by the large number of novel genes of unknown function revealed by genome sequencing and meta-genomic studies [3]. The comparative analysis of bacteriophage genome sequences has greatly enhanced our understanding of their diversity, revealing relationships between phage genomes often infecting distantly related host bacteria. Detailed comparative analyses have been applied to phages infecting hosts including *Bacillus*, *Lactococcus*, *Mycobacterium, Pseudomonas, Salmonella* and *Vibrio* species, marine cyanobacteria, as well as for 337 phages infecting the family Enterobacteriaceae [4,5,6,7,8,9,10,11,12,13,14]. Comparative genomics has revealed the genomes of the dsDNA tailed bacteriophages to be modular. The isolation of phages, closely related at both the nucleotide and protein level, across disparate geographic locations, environments and time indicates that many phage clades are widely distributed and that horizontal gene transfer does not completely mask phage evolution [15,16,17]. Additional selective pressures influencing phage evolution include bacterial immunity systems such as CRISPR (clustered regularly interspaced short palindromic repeats), restriction-modification and abortive infection systems [18,19].

Members of the genus *Acinetobacter* are strict aerobes, non-motile, non-fermentative, catalase-positive and oxidase-negative Gram-negative rods [20]. The genus has undergone extensive taxonomic changes with members historically classified as *Bacterium anitratum* (B5W group), *Herella vaginicola*, *Moraxella glucidolytica*, *Moraxella lwoffii*, *Micrococcus calcoaceticus*, *Mima plomorpha* and *Achromobacter* sp [21]. Today, the genus is a complex and heterogeneous group comprised of 55 species with valid names [22]. A variety of molecular methods are employed for species identification including DNA-DNA hybridisation [23], amplified ribosomal DNA restriction analysis [24], multi-locus sequence typing [25,26] and genome sequence data [27]. The first electron micrographs of *Acinetobacter* phages were published in 1966 and over 100 isolates have since been documented worldwide [28,29,30]. Interest in bacteriophages infecting species of *Acinetobacter* has increased in recent years, primarily due to the emergence of multi-drug resistant *A. baumannii* as a prominent opportunistic pathogen associated with nosocomial and community-acquired infections [31]. The significance of multiple-drug resistance in *A. baumannii* is reflected by the recent classification of this species as a “Priority 1: Critical” pathogen in the World Health Organisation list of pathogens for the research and development of new antibiotics [32]. Infections associated with *A. baumannii* include ventilator-associated pneumonia, soft tissue infections associated with wounds and burns, bacteraemia, urinary tract infections and secondary meningitis [33]. Notably, other *Acinetobacter* species are being associated with nosocomial infections with increasing frequency and have been suggested to represent emerging pathogens [34].

This work presents a review of the *Acinetobacter* phages sequenced to date, expanding upon previous classifications to encompass new phage isolates using available genomic and morphological data [29]. To investigate their genetic diversity, 37 sequenced *Acinetobacter* phage genomes were re-annotated and examined using a comparative bioinformatics approach based upon whole genome alignments, protein clustering and phylogenetic analysis.

## 2. Materials and Methods

Whole genome sequences were downloaded from GenBank (Table 1). Five *Acinetobacter* prophages isolated and sequenced following induction from their bacterial host were deliberately excluded from this study. Searches were performed using BLASTn (megablast and dc-megablast) and tBLASTx with an E-value cut-off of 0.1 for each phage to identify similar phage genomes deposited in the international sequence databases [35]. Nucleotide dot-plots were prepared using Gepard [36] and average nucleotide identity (ANI) was calculated using the BLASTn algorithm in JSpecies 1.2.1 [37].

Phage genomes were re-annotated using a combination of GeneMark, Glimmer and Prodigal to identify open reading frames (ORFs) [38,39,40]. Where the ORF predictions did not agree, start sites were chosen after manual inspection of upstream sequences for putative ribosome binding sites and evidence obtained from searches using BLASTp. Functional inferences for ORFs encoded by all phages were obtained from searches of the non-redundant database using BLASTp with an e-value cut-off of 0.1 as well as conserved domains and motifs identified using pfam_scan.pl against the Pfam31 database and InterProScan 5.25-64 [41,42]. Translated ORF sequences were also searched against hidden Markov model profiles downloaded from the prokaryotic Virus Orthologous Groups database [43] using hmmscan [44] with an e-value cut-off of 1 × 10^−3^. Matches to pVOG profiles were considered significant at an e-value of ≤ 1 × 10^−15^ and ≥35% coverage of the profile HMM. Putative rho-independent terminators were predicted using ARNold and TransTermHP v2.0.9, respectively [45,46]. tRNAScan-SE and ARAGORN were used to predict tRNAs [47,48].

For the identification of orthologous groups of proteins, translated ORF sequences were clustered into groups using OrthoMCL with an e-value of 1e^−5^ and an inflation value of 1.15 [49]. The OrthoMCL matrix was converted to a binary matrix such that the presence and absence of a gene was denoted as 1 and 0, respectively, for each phage isolate. This matrix was used to calculate Jaccard distances between each phage and was subsequently converted to Nexus format using the phylogeny.fr format converter [50,51] and loaded into SplitsTree [52]. Proteins grouped by OrthoMCL were aligned using Clustal Omega [53] and these alignments were then used to search the uniprot20_2015_06 database using HHblits with one iteration and an e-value threshold of 0.001 [54]. Hidden Markov models were constructed from the expanded HHblits alignments produced using HHmake and then queried against the pdb70_from_mmcif_05July17 database using HHsearch. Remote homologies identified using HHsearch were used to further refine the phage genome annotations. Comparisons of conserved gene product content between the *Acinetobacter* phages and the wider sequenced phage pool were performed using CoreGenes3.5 [55]. Genome maps were prepared in Scalable Vector Graphics format using the CGView Comparison Tool [56] or EasyFig [57] and edited using Adobe Illustrator.

Phage transmission electron micrographs were provided courtesy of members of the phage research community (c.f. Acknowledgements) or are reproduced with the permission of the respective publishers. The micrograph of phage 133 was reproduced from Archives of Virology, A catalogue of T4-type bacteriophages, volume 142, 1997, page 2337, Ackermann, H.-W. & Krisch, H.M. (© Springer-Verlag 1997) with permission of Springer. The micrograph of phiAB2 was reproduced from Lin et al. Isolation and characterization of ϕAB2: a novel bacteriophage of *Acinetobacter baumannii*. Research in Microbiology 2010; 161(4):308–314. Copyright © 2010 Elsevier Masson SAS. All rights reserved. The micrograph of R3177 was reproduced from Archives of Virology, Complete genome sequence of the siphoviral bacteriophage Βϕ-R3177, which lyses an OXA-66-producing carbapenem-resistant *Acinetobacter baumannii* isolate, volume 160, 2015, page 3158, Jeon et al. (© Springer-Verlag Wien 2015) with permission of Springer. Micrographs of IME-AB2 and Acibel007 were reproduced from [58,59], respectively, under the terms of the Creative Commons Attribution license.

## 3. Results

### 3.1. Acinetobacter Phages with Whole Genome Sequences

Bacteriophages are classified by the International Committee on Taxonomy of Viruses (ICTV) according to phenotypic and genotypic parameters that encompass virion morphology, nucleic acid type, genome organisation, nucleotide sequence identity, number of shared proteins and phylogenetic analysis of encoded proteins [60]. With the exception of the ssRNA Levivirus AP205, the sequenced phages infecting *Acinetobacter* spp. thus far belong exclusively to clades within the three families of the Order *Caudovirales*; the *Myoviridae, Podoviridae* and *Siphoviridae* (Table 1).

### 3.2. Whole Genome and Proteome Comparisons of the Acinetobacter Phages

**Figure 1:**
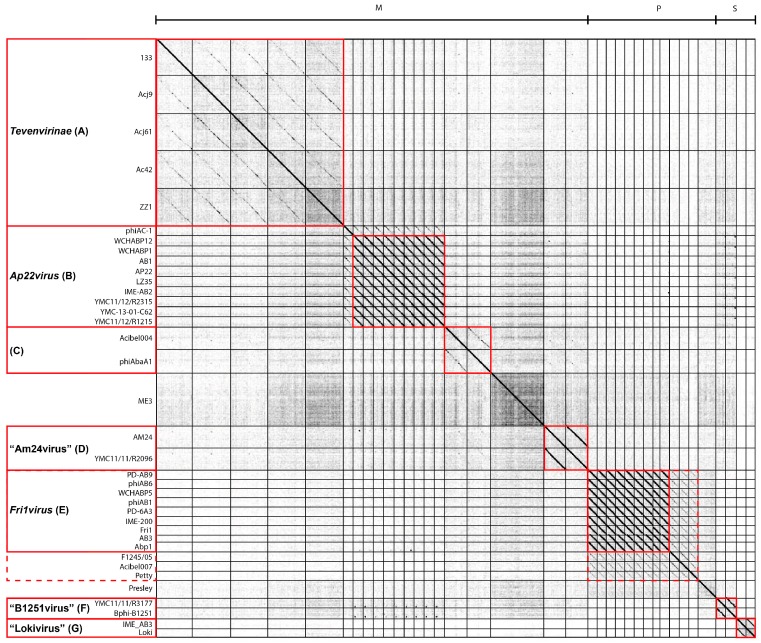
Dot plot alignment of nucleotide sequences from 37 *Acinetobacter* phage genomes. A FASTA file was constructed by concatenating all 37 *Acinetobacter* phage genomes. Where necessary, genomes were reverse complemented and/or colinearized. The 2,655,181 bp file was compared to itself using Genome Pair Rapid Dotter (GEPARD) [36]. Black diagonal lines parallel to the main diagonal indicate strong and continuous sequence similarity while grey lines indicate weaker sequence relationships due to interruptions that result in a discontinuous line. Vertical and horizontal lines were added to indicate individual phage genomes and red boxes to illustrate the assignment of clusters. The dashed red box denotes *Acinetobacter* phages belonging to the podovirus subfamily *Autographivirinae*. The phage family is shown on the horizontal axis (M, *Myoviridae*; P, *Podoviridae*; S, *Siphoviridae*) and the name of each phage is denoted on the vertical axis. Clusters corresponding to existing ICTV taxons and newly proposed genera are labelled.

To visualize relationships between the sequenced *Acinetobacter* phages, the phage genomes were analysed using whole genome dot plots, pairwise sequence identity and shared proteins. Dot plots and nucleotide sequence alignments after genome co-linearization indicate that while there is substantial diversity among the sequenced *Acinetobacter* phages, there is also sufficient similarity to allow these phages to be placed in six discrete clusters designated A–G (Figure 1 and Figure S1). Since several phages exhibited a significant proportion of shared gene content without having substantial nucleotide identity, clusters were defined on the basis of at least 40% of shared gene content.

**Figure 2:**
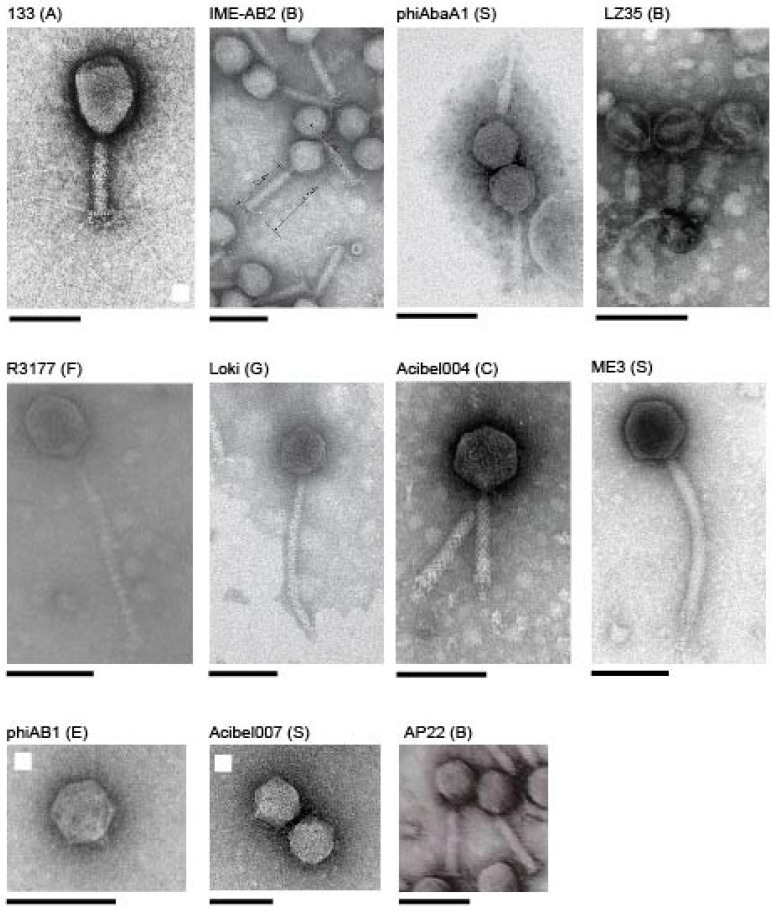
*Acinetobacter* phage morphologies. Available electron micrographs of *Acinetobacter* phages representing clusters and genomic singletons. The name of the corresponding phage is labelled above each micrograph with the designated cluster in parenthesis (S; genomic singleton). The scale bars positioned below each micrograph correspond to 100 nm. Electron micrographs of phages 133, IME-AB2, R3177, phiAB1 and Acibel007 are reproduced with permission from [58,59,83,86,87].

With the exception of clusters A and C, each cluster possesses a minimum of 40% ANI and greater than 50% conservation at the protein level (Figure S2). This division is further supported by morphological similarity (Figure 2) and by low standard deviation in genome size, G + C content and the number of genes encoded between members of each group (Table 1). Three of the proposed clusters correspond to ICTV taxonomic assignments: Cluster A comprises phages related to members of the *Tevenvirinae* subfamily while two clusters, B and E, correspond to the formally established genera *Fri1virus* and *Ap22virus*. Two phages, ME3 and Presley did not reveal a clear relationship to the other phages and are designated as genomic singletons. Several phages exhibited low ANI to the defined clusters. Specifically, phiAC-1 exhibits ANI ranging from 16.2% to 21.4% with phages of the *Ap22virus* (Cluster B) while phages F1245/05, Acibel007 and Petty possess between 9.3% and 25.6% ANI with phages assigned to the *Fri1virus* (Cluster D).

**Figure 3:**
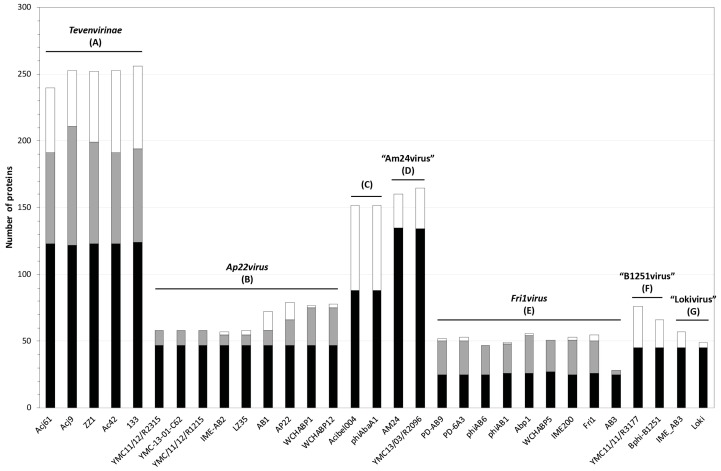
Summary of core, accessory and unique protein groups of individual phage clusters. Core proteins, those encoded by all phages within a cluster, are shown in black. Accessory proteins, those present in two or more but not all phages within a cluster, are shown in grey while unique proteins are shown in white. Clusters comprised of just two phages have no accessory protein count. Phages designated as genomic singletons are not displayed. Clusters corresponding to existing ICTV/proposed genera have been labelled.

The comparison of gene content provides a secondary assessment of genomic diversity. During a manual pairwise comparison, it was observed that some candidate ORFs had not been identified and others had optimal ribosome binding sequences that differ from the published annotations. For these reasons, each phage was re-annotated prior to protein clustering (File S1). Initially, proteins from the five clusters were assembled separately into groups using OrthoMCL. From these results, sets of proteins were identified for each cluster of phages that (i) were present in all genomes within that cluster, (ii) were encoded on two or more but not all, of the genomes in a cluster and (iii) proteins that were unique to a single phage genome (Figure 3).

**Figure 4:**
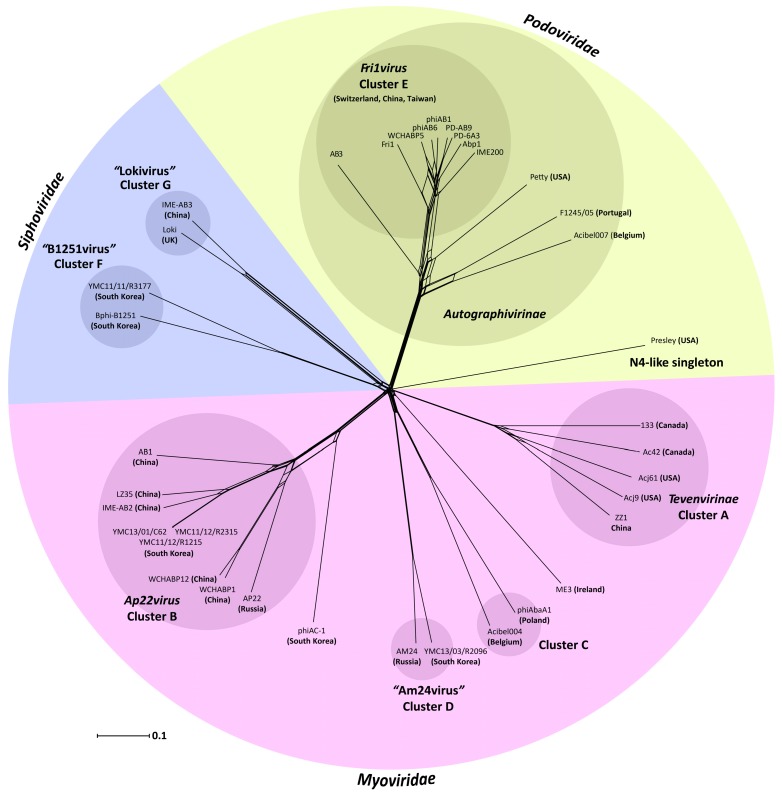
Split graph illustrating the results of Neighbour Net analysis obtained on Jaccard distances for 4065 genes encoded by 37 *Acinetobacter* phages. The network was generated in SplitsTree 4.3 [78], using the neighbour net method upon a Jaccard distance matrix calculated from gene presence or absence. The node labels represent individual phage genomes and their country of isolation (listed in Table 1). The phage clusters recognized by the ICTV or identified by nucleotide sequence identity (Figure 1.) are annotated. The scale bar represents number of gene differences (present or absent) per gene site. The circular wedges are coloured and labelled according to the phage family. Labels in quotation marks correspond to new genera proposed as a result of this work.

To estimate the gene content relationship between all 37 phages, OrthoMCL was used to cluster all 4065 proteins, which assembled into 737 groups of two or more proteins and 975 orphans (File S2). This approach allowed the inter- and intra-cluster relationship in gene content amongst the *Acinetobacter* phages to be represented as a network phylogeny, the results of which show agreement with the clusters designated from nucleotide sequence comparisons (Figure 4). Highlighting their status as singletons, 260 and 80 of the proteins encoded by ME3 and Presley, respectively, were unique orphans. The relationship between the more distantly related phages is more apparent from the gene content analysis where Petty, F1245/05 and Acibel007 each encode 22 proteins (41.5% to 48.9% of ORFs) that represent core protein groups of the *Fri1virus*. Similarly, 29 proteins (35.4% of ORFs) encoded by phiAC-1 fall within the core *Ap22virus* protein groups. These more distant relationships are represented by common branches in the network phylogeny (Figure 4)*.*

Functional inferences for the grouped and unique protein sequences were obtained using a combination of BLASTP, InterProScan and the HHsuite tools, HHblits and HHsearch. Of the 4067 proteins, a putative function based on bioinformatics analysis could be ascribed to 1762 (322 protein groups and 172 orphans) while 2305 (56.6%) were annotated as hypothetical proteins of unknown function. The majority of proteins formed mutually exclusive groups common to two or more members of a single cluster (File S2).

A total of 37 core and three accessory protein groups were shared between two clusters of phages, predominantly between the myoviruses. Clusters C and D shared the greatest number of protein groups that include 17 virion structural and assembly proteins in addition to a predicted thioredoxin, endodeoxyribonuclease, replicative helicase and DNA polymerase. Each of the T4-like and Fri1-like phages encode a predicted ATP-dependent ATP ligase and deoxynucleotide monophosphate (dNMP) kinase that group together in the OrthoMCL analysis. Three core structural proteins are shared between the *Acinetobacter* siphoviruses; the predicted portal vertex protein, major capsid protein and a putative tail completion protein.

The three accessory protein groups shared between clusters correspond to a hypothetical protein (clusters A and E), a HNH family homing endonuclease (clusters B and E) and a glycoside family 24 endolysin (clusters A and B). The examination of pairwise comparison maps indicates that inclusion of accessory and unique orphan genes in some phages are not uniformly distributed across the genome. The genomic modules containing genes responsible for the assembly of virion structure, nucleic acid metabolism and genome replication tend to be highly conversed in both gene content and order. Despite the comparisons in gene content defining sharp boundaries between the clusters, there are examples of mosaicism both within and between clusters that are particularly apparent in the tailspike and endolysin genes.

One of the major determinants of host specificity, phage receptor binding proteins appear subject to relatively frequent recombination events [88,89]. Twenty-five phages representing Clusters B–D and the singleton ME3 were predicted to encode tailspikes adopting a parallel β-helix structure (File S2). Tailspike proteins tend to exhibit a bimodular structure consisting of an N-terminal virion binding domain and a C-terminal receptor binding domain that often possesses enzymatic activity [90]. While the N-terminal tailspike sequence is both highly conserved and specific to each cluster of *Acinetobacter* phages there appears to be two clear examples of domain mosaicism where the C-terminal sequence is highly conserved between two members of different clusters. Specifically, the C-terminal sequences of the Cluster B myovirus IME-AB2 and Cluster D podovirus Abp1 exhibit 97% identities. The myoviruses WCHABP12 and AM24 also possess highly similar (94% identities) C-terminal sequences but divergent N-termini.

*Acinetobacter* phage endolysins represent a mixture of single and multiple domain endolysins where a lysozyme-like domain is accompanied by a peptidoglycan-binding domain. While the position of the lysis cassette is conserved within each cluster, the endolysins from the 37 phages form four protein groups that do not segregate according to cluster. ME3 appears to have the lysis functions encoded in two separate, adjacent genes with locus tags ME3_7 and ME3_8, encoding a predicted peptidoglycan-binding protein and lysozyme, respectively.

In the following sections, the common characteristics and distinguishing features for each cluster of phages are briefly discussed.

#### 3.2.1. Cluster A: The T4-Like *Acinetobacter* Phages

The *Acinetobacter* phages 133, Acj9, Acj61, Ac42 and ZZ1 all belong to the large and ubiquitous myovirus subfamily *Tevenvirinae*. The T4-like morphology is characterised by an elongated icosahedral capsid and a contractile tail exhibiting transverse striations and a collar [86]. The tail terminates in a baseplate that carries six long kinked tail fibers and six short tail spikes. In the quiescent state, the long tail fibers are held in a folded configuration by whisker fibers extending from the collar. With the exception of ZZ1, whose dimensions were obtained without magnification control, all the *Acinetobacter* T4-like phages exhibit similar dimensions and consist of a moderately elongated head of 120 by 86 nm, a contractile tail identical in morphology to T4 of 111 by 16 nm terminating in a baseplate with long and short tail fibers [86]. The five sequenced *Acinetobacter* T4-like phages encapsulate a 159 to 169 kbp genome encoding between 241 and 257 ORFs (Table 1) and exhibit low intra-cluster ANI of between 10 and 26 %.

Previous studies have demonstrated that the T4-like phages comprise a core genome which consists of genes involved in DNA replication, virion structural components and assembly chaperones [62]. Each of the *Acinetobacter* T4-like phages share 122 conserved core proteins and between 68 and 89 proteins that form accessory protein groups. Gene products unique to each phage occupy approximately 20% of the genome. Relative to other members of the *Tevenvirinae*, CoreGenes analysis indicates that >40% of the gene products have a homolog encoded by T4, RB69, RB49, JS98, SP18, JD18 and CC31 (File S3).

The presence of homologs to Alt, MotA, AsiA and gp55 in the Cluster A *Acinetobacter* phages suggest the regulation of transcription follows a similar pattern to T4. Alt is an internal head protein injected with the T4 genome with mono-ADP-ribosyltranferase activity that modifies the bacterial RNA polymerase enhancing transcription of early T4 RNAs. The middle transcription activator MotA binds to the MotA box to activate transcription in the presence of AsiA-associated host RNA polymerase [91]. Gp55 is a sigma70 family protein that binds to the host RNA polymerase and facilitates recognition of the late promoter sequence motif [92].

The low level of sequence identity exhibited between the T-like *Acinetobacter* phages confirms that each represents a distinct T4-like species. With the isolation of related phages, the T4-like *Acinetobacter* phages may form further genera to the 11 already established within the *Tevenvirinae* subfamily but for the time being represent unclassified species [62,93].

#### 3.2.2. Cluster B: The AP22-Like *Acinetobacter* Myoviruses (*Ap22virus*)

**Figure 5:**
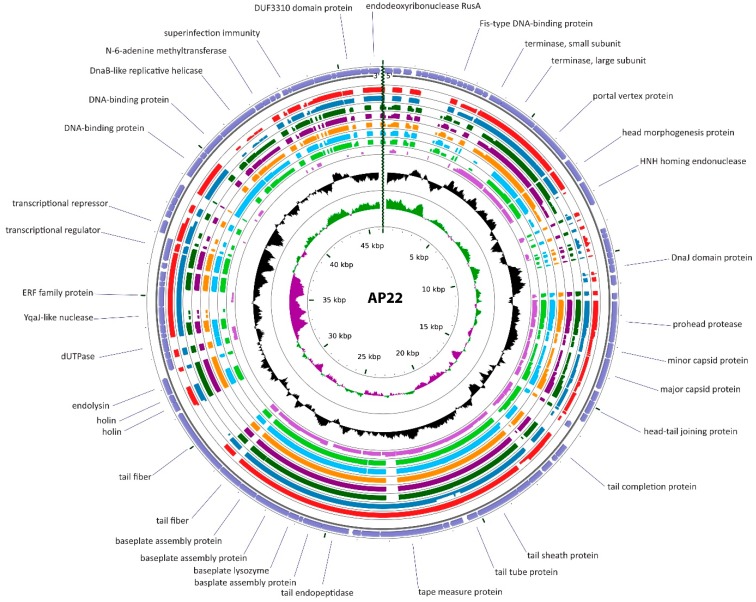
Comparative genome map of AP22 and the Cluster B phages. AP22 was used as the reference genome due to its designation as the type species of the genus Ap22virus. The inner rings display tBLASTx comparisons to WCHABP12 (red), WCHABP1 (dark blue), YMC-11/12/R2315 (dark green), YMC-13-01-C62 (dark purple), YMC-11/12/R1215 (orange), LZ35 (light blue), IME-AB2 (light green) and phiAC-1 (light purple). GC content is depicted in black while positive and negative cumulative GC skew is shown as green and purple, respectively.

Cluster B is comprised of nine *Myoviridae;* AP22, AB1, IME-AB2, LZ35, YMC-13-01-C62, YMC11/12/R2315, YMC11/12/R1215, WCHABP1 and WCHABP12 that encapsulate genomes of between 43.2 and 46.4 kbp and encode between 82 and 89 ORFs (Table 1, Figure 5). Three of these phages have been examined by electron microscopy. Micrographs of IME-AB2 and AB1 show a single morphotype consisting of an isometric head and a contractile tail terminating in a baseplate with short tail fibers [58,68]. This grouping has been recognized independently and an ICTV proposal to create a new genus within the family *Myoviridae*, the *Ap22virus* was ratified in 2016 [93]. These phages share at minimum 40% ANI and share between 61.5 and 100% proteins. We note that YMC-13-01-C62, YMC-11/12/R2315 and YMC11/12/R1215 have average nucleotide sequence identities of 99%, which is above the current ICTV species demarcation criteria of 95% indicating that they should be considered as isolates from the same phage species [94]. The AP22-like genomes exhibit a modular and syntenic organization with the majority of genes encoded on the forward strand. In each of these phages, the putative head morphogenesis protein is separated from a predicted prohead protease by between seven and 13 ORFs of unknown function. The endolysin and holin are encoded at the end of the structural and assembly gene module, followed by two modules encoding genes involved in nucleotide metabolism, recombination and superinfection immunity (Figure 5). The *Ap22virus* core genome consists of 47 gene products that include virion structural and assembly proteins, a superinfection immunity protein, SaV-like domain protein, primase/helicase and transcriptional regulatory proteins.

While members of the *Ap22virus* show little nucleotide similarity to other phages in the extant sequence database, a small number of myoviruses infecting the genera *Aeromonas* (51 and vB_AsaM-56), *Burkholderia* (Bcep1, Bcep43 and Bcep781), *Edwardsiella* (GF-2) and *Xanthomonas* (OP2) were identified as encoding protein homologs by tBLASTx. CoreGenes comparisons show these phages possess between 19 and 27 homologs (File S2) to the virion structural and assembly genes of the *Ap22virus*.

Phage phiAC-1 is clearly more distantly related to members of the *Ap22virus*, exhibiting greatest nucleotide sequence identity with AP22 at 21.4%. This feature is reflected by the OrthoMCL analysis where 34 (41.5%) phiAC-1 proteins are designated as unique orphans and these differences are apparent in the visual comparative analysis (Figure 5). In contrast to the other phages comprising cluster B, all of which infect strains of *A. baumannii*, phiAC-1 is propagated upon *Acinetobacter soli* strain KZ-1 and is reported to show a narrow host range [73]. Functions could be predicted for only seven of the 34 orphan phiAC-1 proteins, which include a tailspike protein, single-stranded DNA-binding protein, YqaJ/RecB-like exonuclease, phosphoadenylyl sulphate reductase, a putative helicase loader and two proteins with a predicted EF-hand domain and a domain of unknown function (DUF1376), respectively.

#### 3.2.3. Cluster C: Myoviruses Acibel004 and PhiAbaA1

vB_AbaM_Acibel004 has a 99.7 kbp genome encoding 156 ORFs and 22 tRNAs with genes organised into several functional modules that include a lysis cassette, DNA packaging, virion structure and assembly, nucleic acid metabolism, genome replication and tRNA modules. The virion structure consists of an isometric capsid 70 nm in diameter and a 105 nm long contractile tail [59], which in the quiescent state appears to show a triangular cluster of tail fibers at the tail terminus (Figure 2). The structure appears to form a hexagonal pyramid with the fibers meeting at an apex. This structure is also evident in micrographs of phiAB11 and Abp53 and appears to be lost upon contraction of the tail. Twenty-six proteins were identified from analysis of Acibel004 virions by electrospray ionisation mass spectrometry and a number show similarity to the putative structural proteins of *Pseudomonas* phages PAK-P1, PAK-P3 and KPP10 [59], a relationship that is confirmed by CoreGenes analysis (File S3). Acibel004 possesses 20.5% ANI and 56.4% of shared proteins with phiAbaA1. A further tentative relationship can be found for the 10,290 bp partial sequence available for Abp53 [JF317274.1], a phage of similar morphology to Acibel004 [95] and which is annotated to include several virion structural genes. The partial Abp53 sequence shows 71% identity across 4% of the Acibel004 genome by dc-megablast. Lastly, we note that Lin et al. [87] have described an additional *Acinetobacter* phage of similar morphology, phiAB11.

#### 3.2.4. Cluster D: Myoviruses AM24 and YMC13/03/R2096

The grouping of phages AM24 and YMC13/03/R2096 into Cluster D is supported by ANI of 75.4% and a total of 134 proteins shared across the two genomes. The AM24 and R2096 genomes encode 18 and 17 tRNAs in addition to 168 and 170 ORFs, respectively (Table 1). Both phages exhibit an almost identical organization consisting of four modules. No putative small terminase subunit could be identified within the DNA packaging and virion structural and assembly gene module. An additional tail fiber and SleB-like cell wall hydrolase are encoded upstream of the large terminase subunit, separated by a cluster of tRNAs. Both phages are replete with ORFs encoding proteins of unknown function, accounting for approximately 70% of all genes.

No related phages within the wider sequenced population were identified by searches conducted with dc-megablast. However, CoreGenes comparisons of phages identified by TBLASTX demonstrate that these two phages share a maximum of 20% of proteins with members of the *Pakpunavirus* and the *Pseudomonas syringae* phages KIL1, KIL2, KIL3, KIL4 and KIL5 (File S3). The shared gene products are predominantly involved in virion structure and assembly but also include those involved in nucleotide replication and metabolism. Based on the nucleotide identity and shared gene content of these two phages, the creation of a new genus within the Family *Myoviridae* is proposed, named the “R2096virus” after the first isolated member.

#### 3.2.5. Cluster E: *Acinetobacter* Phages of the Subfamily *Autographivirinae*; Genus *Fri1virus*

The Cluster E *Acinetobacter* phages represents the recently established genus *Fri1virus* within the subfamily *Autographivirinae* [93] and is comprised of nine podoviruses; AB3, Abp1, IME-200, phiAB1, phiAB6, PD-AB9, PD-6A3, WCHABP5 and the type species Fri1 (Table 1). Like other phages of the *Autographivirinae,* the Fri1-like viruses all encode their own single subunit RNA polymerase (RNAP) and share a common overall genomic organization with genes encoded solely on the forward strand [84]. Alongside the *Fri1virus* six other genera, the *Phikmvvirus*, *Sp6virus*, *Kp32virus*, *Kp34virus*, *Pradovirus* and *T7virus* have been defined within the *Autographivirinae*.

**Figure 6:**
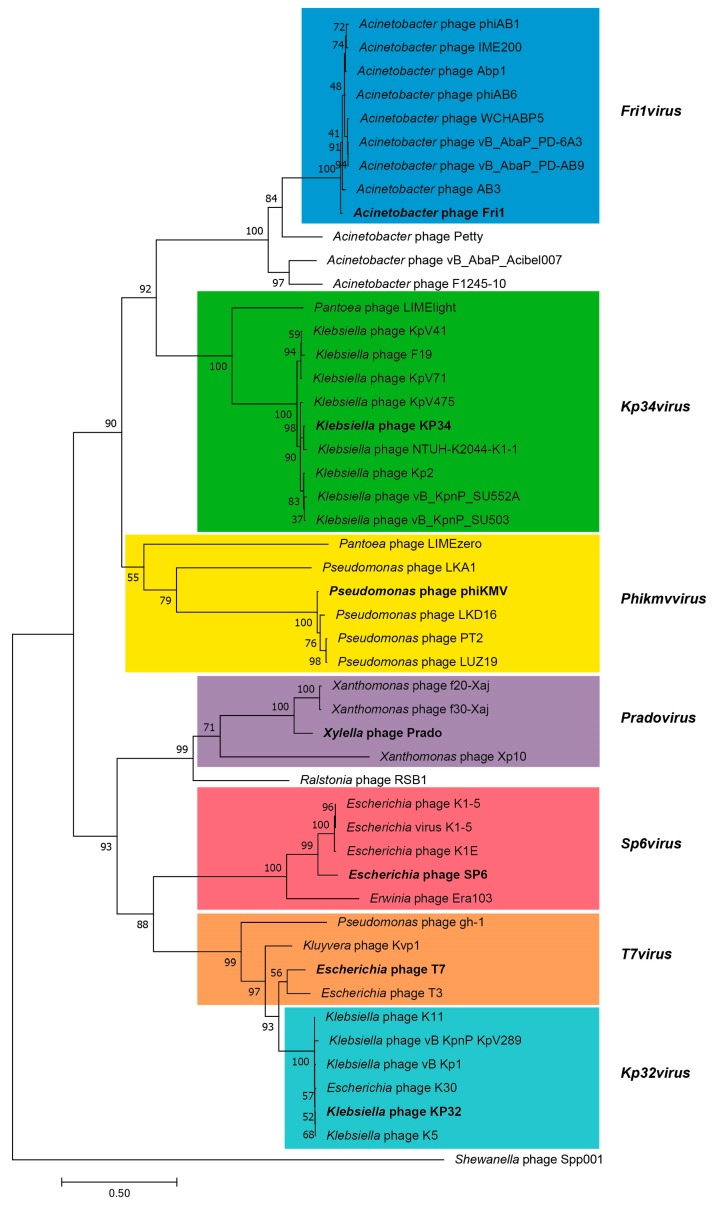
Neighbour-joining tree analysis of the DNA-dependent RNA polymerases of phages comprising ratified ICTV genera within the podovirus subfamily *Autographivirinae*. The tree was rooted using the RNA polymerase from *Shewanella* phage Spp001 [YP_009008879] as the out group. The phylogenetic tree was constructed using a MUSCLE alignment [97] and the maximum likelihood method in MEGA7 [98] with 2000 bootstrap replicates. The bootstrap percentages are shown next to each node. The scale represents the number of amino acid substitutions per site. Phage genera as defined in the ICTV 2016 virus taxonomy release are delineated by coloured blocks with the type species for each genus displayed in bold text.

The Fri1-like phages possess an average genome size and G+C content of 41.7 kbp and 40.15%, respectively. We note that the AB3 genome is missing approximately 10 kbp of sequence compared to its close relatives, corresponding to the left end of the genome [96]. Two truncated ORFs that are not annotated in the associated GenBank entry, present at the right and left ends of the sequence, are predicted to encode a putative DNA maturase B and DNA helicase, respectively [96]. Despite possessing between 40.8% and 34.7% shared proteins relative to members of the genera *Phikmvvirus and Kp34virus* and exhibiting a similar genome organization, there are sufficient differences to separate these phages from previously established genera within the *Autographivirinae*. These phages encode a single subunit RNAP situated adjacent to the structural genes and a class I holin with three predicted transmembrane domains and an endolysin with a glycoside hydrolase family 19 domain (InterPro: IPR000726) situated between the tail fiber and the small terminase subunit. Each phage is predicted to encode two rho-independent transcriptional terminators, located downstream of the RNAP and major capsid proteins. In addition, the recognition and specificity loops of the RNA polymerase are conserved within these viruses and differ substantially from those reported for the *Phikmvvirus* and *Kp34virus* [17]. Phylogenetic analysis of the RNA polymerase demonstrates that these phages fall into a single monophyletic clade (Figure 6).

#### 3.2.6. Cluster F: The 531-Like *Acinetobacter* Siphoviruses

**Figure 7:**
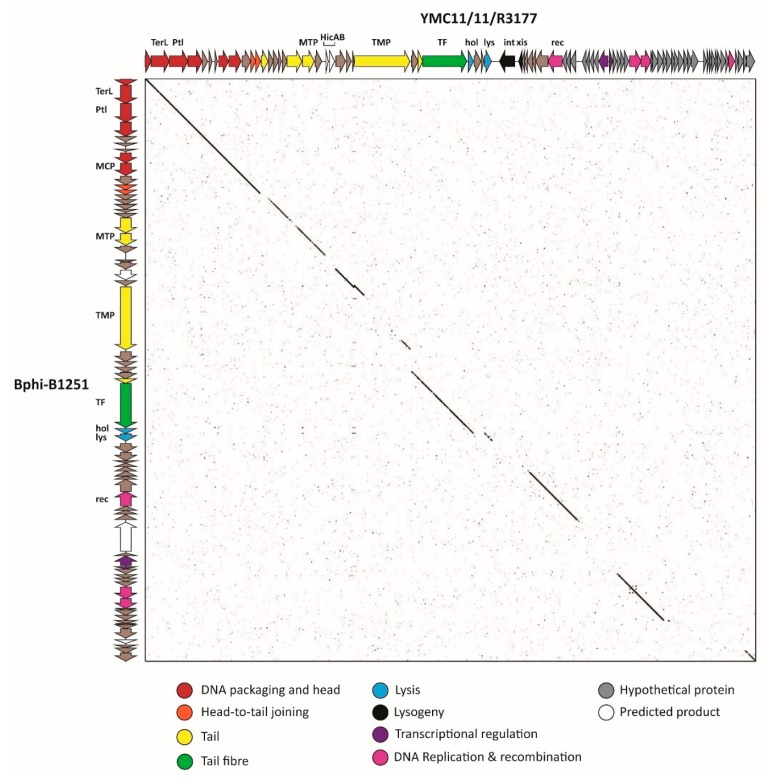
Dot plot comparison of the nucleotide sequences of *Acinetobacter* phages YMC11/11/R3177 and Bphi-B1251 illustrating breaks in syntenic similarity. Genome maps are displayed on the horizontal and vertical axes. Genes identified by bioinformatics analysis as belonging to specific modules are color-coded according to the key. Selected genes are annotated in the figure: TerL, large subunit terminase; Ptl, portal vertex protein; MCP, major capsid protein; MTP, major tail protein; TMP, tape measure protein; HicAB, HicAB toxin-antitoxin; hol, holin; lys, lysin; int, integrase; xis, excisionase; rec, putative RecA-like recombination protein. The figure was constructed using Gepard and EasyFig and edited using Adobe Illustrator.

Cluster F is comprised of two phages, Bphi-B1251 and YMC11/11/R3177 (R3177) that share 61% ANI and 67% protein homologs. Bphi-B1251 encapsulates a 45.4 kbp 39.05% G+C genome encoding 66 ORFs while R3177 encapsulates a larger genome of 47.6 kbp encoding 80 ORFs (Table 1). Bphi-B1251 is described as a podovirus, both in the GenBank accession record and in the associated genome announcement [84]. However, homologues of the structural genes of these phages suggests that both phages are in fact siphoviruses, indicated by the presence of a tape-measure gene of 4.9 and 4.3 kbp in Bphi-B1251 and R3177, respectively. Supporting this assignment, R3177 has recently been confirmed as a temperate member of the *Siphoviridae* and exhibits a 531-like morphology [83]. The 531-like phages exhibit a slightly elongated head of 73 × 59 nm and a 252 nm long tail that is characterised by the presence of multiple transverse disks that gives the tail a segmented appearance [29]. Analysis of R3177 and Bphi-B1251 ORFs using HHsuite allowed for the prediction of additional structural, replication and maintenance proteins. Examining dot plot alignment of Bphi-B1215 and R3177 shows that a number of genomic modules exhibit localised differences (Figure 7). In the virion structural and morphogenesis gene module these non-homologous regions correspond to different head-tail joining proteins and a HicAB-like type II toxin-antitoxin cassette in R3177. Additionally, R3177 encodes an integrase, excisionase and transcriptional regulatory proteins that are absent in Bphi-B1251 indicating that, despite significant nucleotide and proteomic similarity, these two related phages undertake different lifestyles. While few protein homologs in other phages were identified by BLASTp, R3177 exhibits strong sequence similarity (>70% coverage, >95% identity BLASTn) to putative prophage regions in the sequenced genomes of *A. baumannii* strains SSA12, KAB04, KAB02, YU-R612 and NCGM237. It would therefore be of interest to determine whether Bphi-B1251 and R3177 are related to the unsequenced phages 531 and B_9_PP, two prophages of similar morphology induced from *Acinetobacter* isolates HER1032 and HER1096, respectively [29]. The clear similarities between these two phages and lack of relatives among the sequenced phages are sufficient to propose the establishment of a new genus, which we tentatively name the “B1251virus.”

#### 3.2.7. Cluster G: The Loki-Like *Acinetobacter* Siphoviruses

**Figure 8:**
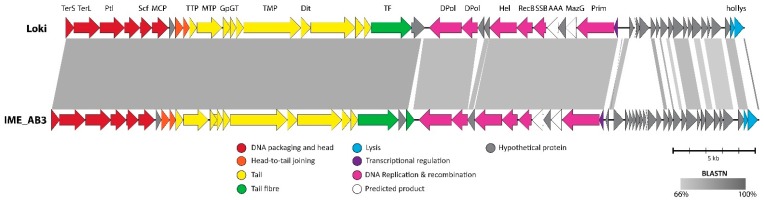
Pairwise BLASTn comparison of the *Acinetobacter* phages Loki and IME_AB3. The pairwise comparison clearly shows that these two phages are primarily distinguished by the constituent genes in the genomic module encoding the lysis cassette. Genes identified by bioinformatics analysis as belonging to specific modules are colour coded according to the key. Selected genes are annotated in the figure: TerS, small terminase subunit; TerL, large subunit terminase; Ptl, portal vertex protein; MCP, major capsid protein; MTP, major tail protein; GpGT, tail assembly chaperones GpG and GpGT; TMP, tape measure protein; Dit, distal tail protein; TF, tail fiber; DPol, DNA polymerase; Hel, helicase; recB, putative RecB-like recombination protein; SSB, single-stranded DNA-binding protein; AAA, AAA ATPase domain protein; hol, holin; lys, lysin. The figure was constructed using EasyFig and edited using Adobe Illustrator.

The two Cluster G phages, vB_AbaS_Loki and IME_AB3 are closely related. Morphologically, Loki is a B1 siphovirus that resembles *Burkholderia* phage vB_BceS_KL1 [99] with an isometric capsid measuring 57 nm across opposite apices and a non-contractile tail that measures 176 nm in length, 10 nm in diameter and exhibits transverse striations [85]. Loki and IME_AB3 encapsulate a genome of 41 and 43 kbp with% mol G+C contents of 44.4 and 45.6%, respectively (Table 1). ANI between the two phages is 58.9% and the 109 encoded ORFs form 45 protein groups and 19 orphans. The genome is organised into three modules containing genes encoding the virion structure and morphogenesis, DNA replication and metabolism and a final module encoding the holin and endolysin that is replete with genes of unknown function (Figure 8). Loki and IME_AB3 are distinguished both by the constituent genes present in this last module and by the structure of the endolysin. The Loki endolysin is modular comprising a lysozyme-like domain (InterPro: IPR023346) and peptidoglycan-binding domains (InterPro: IPR018537), whereas the IME-AB3 endolysin is globular and contains a single lysozyme domain (InterPro: IPR000726). Loki and IME-AB3 each share between 28 and 31 (49.1% to 58.8%) protein homologs with the *Pseudomonas* and *Burkholderia* phages of the *Septima3virus*. These shared proteins represent the majority of genes encoded in the structural and replication gene modules and these phages differ predominantly in the module encoding the endolysin and holin. Due to the similarities in nucleotide sequence, gene content and genome organisation we propose the establishment of a new genus, the “Lokivirus” named after the first fully characterised member.

### 3.3. The Singleton Acinetobacter Phages

#### 3.3.1. Singleton vB_AbaM_ME3

ME3 is classified as a giant or “jumbo” phage with a 234.9 kbp genome that encodes for 326 genes and four tRNAs. The majority of ORFs are encoded on the reverse strand, with only 53 encoded on positive strand and the ME3 genome does not exhibit the modular organisation characteristic of smaller phage genomes. A significant proportion of genes encoded by ME3 are unique among the *Acinetobacter* phages. A total of 260 ME3 proteins (79.8%) were classified as orphans, suggesting that this phage represents a distinct evolutionary lineage. Interestingly there are 11 protein groups that are composed solely of ME3 proteins, perhaps representing an example of gene duplication and divergence. A further 55 proteins cluster with other *Acinetobacter* phages and are predominantly involved in nucleotide metabolism and DNA replication. ME3 encodes a limited number of proteins with homologs in the wider sequenced phage population. Specifically, ME3 shares 42, 33 and 28 proteins with *Cronobacter* phage vB_CsaM_GAP32, *Escherichia* phage 121Q and *Bacillus* phage 0305phi8-36, respectively.

#### 3.3.2. *Acinetobacter* Phage Presley Is an N4-Like Singleton

A single N4-like *Acinetobacter* phage, Presley, has been described [82]. Presley encapsulates a linear 77 kbp genome flanked by direct terminal repeats. A defining characteristic of all N4-like phages are genes encoding for two distinct RNAPs; a giant virion-encapsulated RNAP and a heterodimeric RNAP consisting of small and large subunits [100]. Presley shares 24 (33.3%) protein homologs with N4 that include virion structural proteins, a single strand DNA-binding protein, DNA polymerase, helicase, small and large RNAP subunits (File S3). Notably, like N4 and the N4-like phages of *Pseudomonas aeruginosa* LUZ7, LIT1 and PEV2, the giant virion RNAP of Presley is devoid of cysteine residues, a feature that has been suggested to be of importance in allowing the vRNAP to transit the bacterial membrane [101]. Over 40 N4-like phages have been sequenced and a recent analysis of this group have revealed that they exhibit substantial diversity in terms of nucleotide sequence, gene content as well as genomic organisation [100]. To date four N4-like genera have been established, the *N4virus*, *G7cvirus*, *Luz7virus, Lit1virus* in addition to a pending ICTV proposal to create a further genus, the “Sp58virus.” The diversity exhibited between phages of these genera has led to the suggestion that the N4-like group constitute a higher taxonomic division than subfamily [93,100]. Presley shows little sequence similarity to phages of the established N4-like taxons and exhibits the least number of protein homologues with the other *Acinetobacter* phages. Only 15 proteins encoded by Presley clustered into groups by OrthoMCL with the remaining 80 proteins designated as orphans. Until additional related phages are isolated, Presley remains an N4-like genomic singleton.

#### 3.3.3. Leviviridae

The *Leviviridae* are a family of small single-stranded RNA viruses currently separated into two genera; *Allolevivirus* and *Levivirus*. Two members of the *Leviviridae* infecting *Acinetobacter* have been described; 142 and AP205, of which only the AP205 genome has been sequenced [61]. AP205 virions appear as hexagonal or spherical particles 27–30 nm in diameter under the electron microscope and encapsulate a 4268 bp linear positive-sense ssRNA genome that encodes four proteins; a lysis protein, a maturation protein, a coat protein and an RNA-dependent RNA polymerase. Like all other members of this family characterised to date, AP205 adsorbs to pili to facilitate infection of host cells [61,102].

## 4. Discussion

In this study, we have presented a review of the genetic and morphological diversity of 37 sequenced *Acinetobacter* phages and obtained information through comparative bioinformatics focusing upon genome organisation, putative gene function and shared orthologous proteins. The last review of the *Acinetobacter* phages was published 23 years ago and did not have access to genomic data [29]. Our work has revealed that 37 of the sequenced phages fall into seven distinct clusters and two genomic singletons defined by the criteria of nucleotide sequence identity, shared orthologous genes and genome organisation. We hope that this study will provide an initial framework for the classification of newly isolated *Acinetobacter* phages. Clearly, with the genomes of only 42 *Acinetobacter* phages available, we have not even begun to scratch the surface of a potentially large, diverse and ubiquitous group of phages. It is undoubtable that these clusters and the genetic diversity they represent will be subject to change with the isolation of new *Acinetobacter* phages. The discovery of isolates, either novel or related to the genomic singletons will lead to the establishment of new clades (genera) in the future. Despite the results of protein clustering sharply defining the proposed groupings, each of the *Acinetobacter* phages encodes numerous unique proteins that lack predicted functions. The large number of unique orphan proteins identified here may simply be a result of under-sampling of the *Acinetobacter* phage gene pool. It is possible that the boundaries between these groups of phages will become less defined as more genomes are sequenced, due to the role of horizontal genetic exchange in the evolution of phage genomes. Further work is required to understand the true diversity of these phages and the potential impact they have upon evolution, fitness and virulence of members of the genus *Acinetobacter*. As for other bacterial genera and perhaps indicative of bias within sampling and enrichment procedures, the *Acinetobacter* phages isolated to date overwhelmingly belong to the order *Caudovirales*. The sole exceptions are two representatives of the family *Leviviridae*. However, there is also evidence for the integration of members of the *Inoviridae* (filamentous phages) into the genomes of *A. baumannii* strains AYE and AB0057 [103] and *A. baylyi* ADP1 [104] suggesting that the wider phage pool is more diverse and encompasses more phage families than are currently reflected by the sequenced phages. Moreover, *Acinetobacter* spp. are polylysogenic, playing host to a large and diverse pool of prophages that have yet to be examined in significant detail [105].

The *Acinetobacter* phages are of interest due to their ability to further our understanding of this important opportunistic pathogen and their possible exploitation as genetic tools as has been evidenced for the Mycobacteriophages [106]. Perhaps more urgently, considering the widespread resistance to multiple antibiotics exhibited by species of *Acinetobacter*, an approach to determine the function of *Acinetobacter* phage genes annotated as hypothetical proteins is required. The majority of core protein groups identified for each cluster of phages correspond to genes encoding key products in genome replication or those expressed as late transcripts. However, each of the clusters identified here possess several core and accessory proteins of unknown function, which are presumed to correspond to essential functions for productive infection and represent candidates for further study. Such functional studies are important, not only from a phage biology perspective to elucidate how these phages interact with and influence the host cell to facilitate productive infection but also for the identification of novel mechanisms of action for the development of new genetic tools and antibacterial agents.

## Figures and Tables

**Table 1:** Characteristics of *Acinetobacter* bacteriophage genome sequences deposited in the International Nucleotide Sequence Database. Phages are grouped according to International Committee on Taxonomy of Viruses (ICTV) taxonomic assignments and on the basis of average nucleotide identity and shared gene content.

Phage	Host	Country	Genome Size	No. of ORFs ^#^	% G+C	Accession	Coding%	Genes/kbp	tRNAs	Reference
Family: *Leviviridae*; Genus: *Levivirus*										
AP205	*Acinetobacter sp.* (HER 1424)	Canada	4268	4	43.86	AF334111	90.9	0.937	0	[61]
Family: *Myoviridae*										
Subfamily: *Tevenvirinae* (Cluster A)										
133	*A. johnsonii* (HER 1423)	Canada	159,801	257 (257)	39.67	HM114315	95.7	1.608	16	[62]
Acj9	*A. johnsonii*	USA	169,947	253 (253)	40.03	HM004124	93.3	1.488	19	[62]
Acj61	*A. johnsonii*	USA	164,093	241 (241)	39.01	GU911519	92.5	1.468	12	[62]
Ac42	*Acinetobacter sp.* (HER 1422)	Canada	167,716	255 (255)	36.37	HM032710	94.6	1.52	6	[62]
ZZ1	*A. baumannii* AB09V	China	166,682	256 (256)	34.41	HQ698922	93.6	1.535	8	[63,64]
Genus: *Ap22virus* (Cluster B)										
AP22	*A. baumannii* 1053	Russia	46,387	89 (90)	37.74	HE806280	91.9	1.918	0	[65,66]
AB1	*A. baumannii* KD311	China	45,159	88 (88)	37.69	HM368260	93.8	1.948	0	[67,68]
IME-AB2	*A. baumannii* MDR-AB2	China	43,665	82 (82)	37.5	JX976549	93.4	1.877	0	[58]
YMC-13-01-C62	A. baumannii YMC/13/01/C62	South Korea	44,844	84 (87)	37.6	KJ817802	94.3	1.94	0	[69]
YMC11/12/R2315	*Acinetobacter sp.*	South Korea	44,846	85 (87)	37.59	KP861229	94.0	1.94	0	[70]
YMC11/12/R1215	*Acinetobacter sp.*	South Korea	44,866	85 (87)	37.59	KP861231	94.3	1.94	0	[70]
WCHABP1	*A. baumannii*	China	45,888	89 (91)	37.63	KY829116	93.9	1.939	0	Unpublished
WCHABP12	*A. baumannii*	China	45,415	88 (91)	37.58	KY670595	94.1	1.937	0	Unpublished
LZ35	*A. baumannii*	China	44,885	83 (85)	38.36	KU510289	92.6	1.782	0	[71]
Unclassified myoviruses (Cluster C)										
Acibel004	*A. baumannii* 070517/0072	Belgium	99,730	156 (156)	37.27	KJ473422	89.2	1.564	22	[59]
vB_AbaM_phiAbaA1	*A. baumannii* Acb8/09	Poland	104,906	165 (165)	37.77	KJ628499	90.7	1.572	13	Unpublished
Proposed genus: “Am24virus” (Cluster D)										
AM24	*A. baumannii*	Russia	97,139	146 (168)	37.25	KY000079	80.4	1.503	17	Unpublished
YMC13/03/R2096	*A. baumannii* YMC13/03/R2096	South Korea	98,170	162 (170)	37.04	KM67266	87.3	1.731	17	Unpublished
Singleton myoviruses										
vB_AbaM_ME3	*A. baumannii* DSM 30007	Ireland	234,900	326 (326)	30.76	KU935715	94.1	1.387	4	[72]
phiAC-1	*A. soli* KZ-1	South Korea	43,216	82 (82)	38.48	JX560521	93.1	1.897	0	[73]
Family: *Podoviridae*										
Subfamily: *Autographivirinae;* Genus: *Fri1virus* (Cluster E)										
Fri1	*A. baumannii 28*	Switzerland	41,805	54 (55)	39.29	KR149290	92.5	1.291	0	Unpublished
Abp1	*A. baumanni* AB1	China	42,185	57 (57)	39.15	JX658790	93.4	1.351	0	[74]
phiAB1	*A. baumannii* M68316	Taiwan	41,526	46 (49)	39.09	HQ186308	91.8	1.179	0	[75]
AB3 *	*A. baumannii*	China	31,185	27 (29)	39.18	KC311669	96.4	0.929	0	[76]
WCHABP5	*A. baumannii* WCHAB1334	China	40,409	47 (52)	39.38	KY888680	88.2	1.163	0	Unpublished
phiAB6	*A. baumannii* 54149	Taiwan	40,570	45 (47)	39.47	KT339321	90	1.109	0	[77,78]
IME200	*A. baumannii*	China	41,243	52 (53)	39.73	KT804908	88.6	1.26	0	Unpublished
vB_AbaP_PD-6A3	*A. baumannii*	China	41,563	48 (53)	39.92	KT388102	92.8	1.154	0	Unpublished
vB_AbaP_PD-AB9	*A. baumannii*	China	40,938	48 (52)	39.34	KT388103	92.9	1.172	0	Unpublished
Unclassified members of Subfamily *Autographvirinae*										
F1245/05	*Acinetobacter sp.*	Portugal	43,016	0 (53)	40.46	HH777814	94.3	1.255	0	[79,80]
Acibel007	*A. baumannii* 070517/0072	Belgium	42,654	53 (53)	41.7	KJ473423	93.9	1.242	0	[59]
Petty	*A. baumannii* AU0783	USA	40,739	45 (45)	42.19	KF669656	91.5	1.104	0	[81]
Unclassified N4-like virus										
Presley	*A. baumannii* M2	USA	77,792	95 (95)	37.77	KF669658	95.4	1.221	0	[82]
Family: *Siphoviridae*										
Proposed genus “B1251virus” (Cluster F)										
YMC11/11/R3177	*Acinetobacter sp.*	South Korea	47,575	80 (81)	39.83	KP861230	90.7	1.681	0	[83]
Bphi-B1251	*A. baumannii* YMC/09/02/B1251	South Korea	45,364	62 (67)	39.05	JX403940	93.5	1.454	0	[84]
Proposed genus: “Lokivirus” (Cluster G)										
IME-AB3	*A. baumannii*	China	43,050	57 (58)	45.48	KF811200	97.1	1.347	0	Unpublished
vB_AbaS_Loki	*A. baumannii* ATCC 17978	UK	41,308	51 (51)	44.35	LN890663	96.4	1.234	0	[85]

* Partially sequenced. ^#^ Numbers in parentheses indicate the total number of open reading frames identified during re-annotation.

## Supplementary Materials

The following are available online at www.mdpi.com/1999-4915/10/1/5/s1, Figure S1: Average nucleotide identity matrix for the *Acinetobacter* phages with sequenced genomes. Data are presented as ANI factored by coverage. Figure S2: Percentage of shared proteins between 37 sequenced *Acinetobacter* phages. File S1: GenBank flat files of reannotated *Acinetobacter* phage genomes. File S2: Distribution and predicted function of protein groups and orphans. File S3: CoreGenes 3.5 proteomic comparisons of the *Acinetobacter* phages with phages infecting other bacterial hosts.
