# Supplementary figures and images for: Comparative Analysis of 37 Acinetobacter Bacteriophages
Source: Viruses. 2017 Dec 24;10(1):5. doi: 10.3390/v10010005 (PMC5795418; doi:10.3390/v10010005)

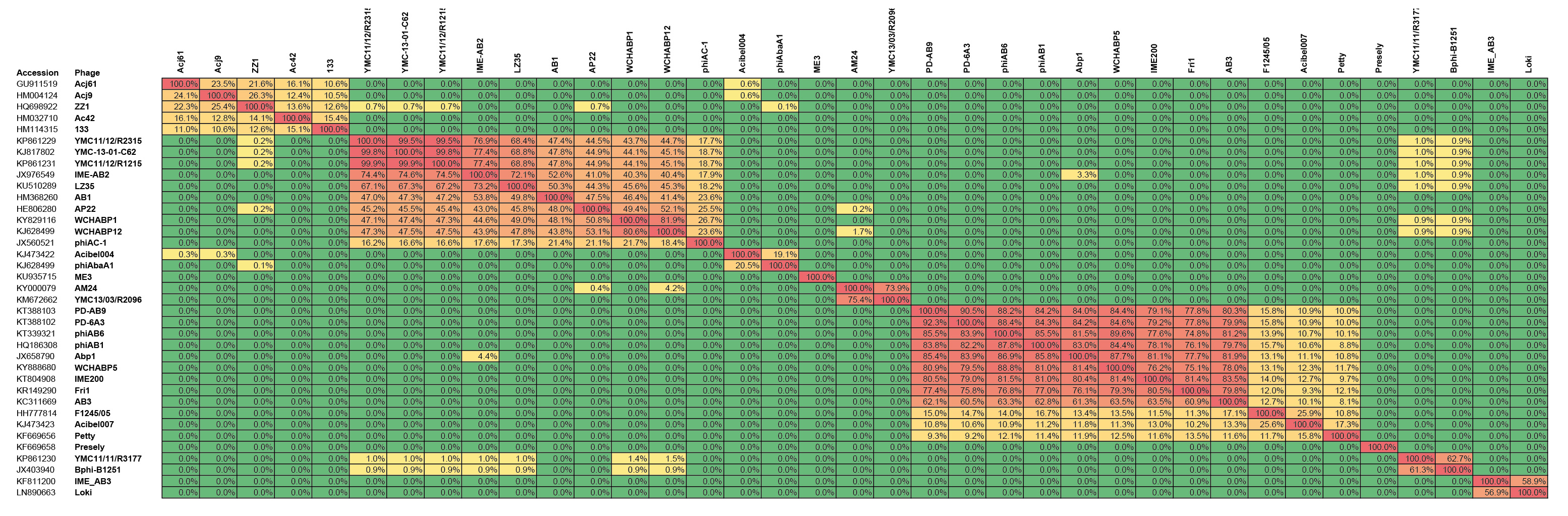

Supplement: Supplementary file 1 [file viruses-10-00005-s001.zip › S1_Figure.jpg]

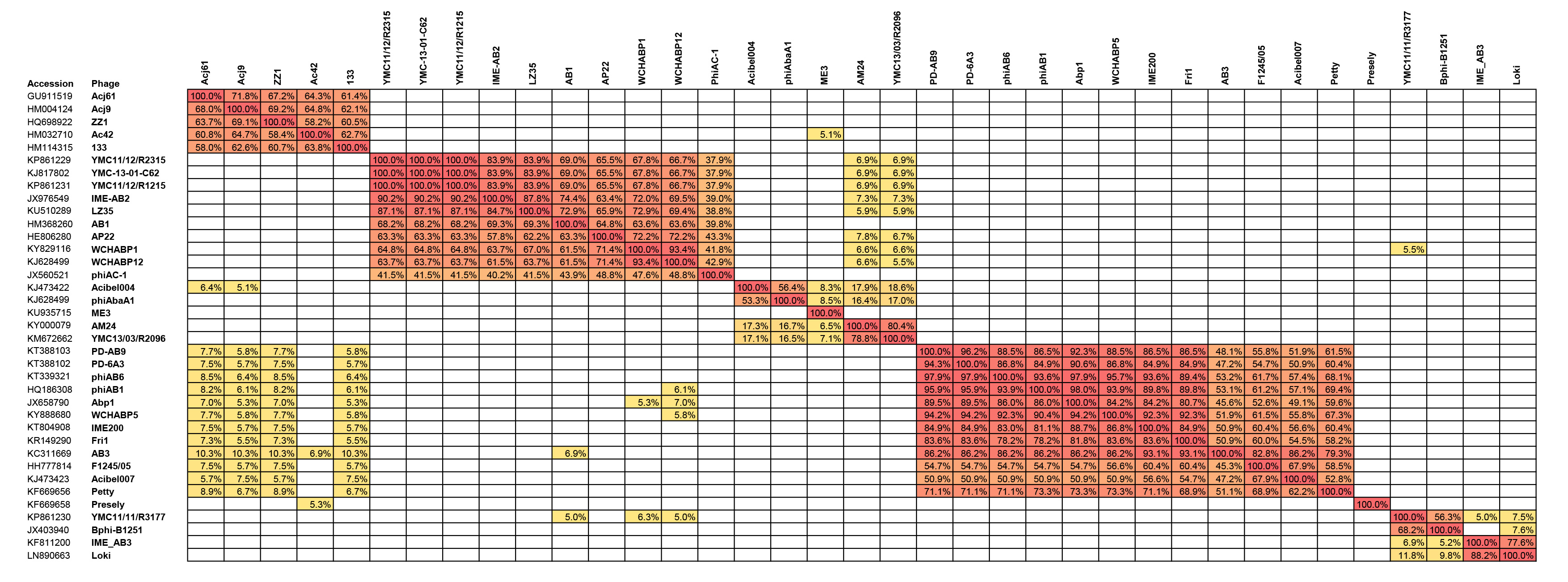

Supplement: Supplementary file 1 [file viruses-10-00005-s001.zip › S2_Figure.jpg]
